# Multi-Task Learning-Based Immunofluorescence Classification of Kidney Disease

**DOI:** 10.3390/ijerph182010798

**Published:** 2021-10-15

**Authors:** Sai Pan, Yibing Fu, Pu Chen, Jiaona Liu, Weicen Liu, Xiaofei Wang, Guangyan Cai, Zhong Yin, Jie Wu, Li Tang, Yong Wang, Shuwei Duan, Ning Dai, Lai Jiang, Mai Xu, Xiangmei Chen

**Affiliations:** 1 National Clinical Research Center for Kidney Diseases, State Key Laboratory of Kidney Diseases, Institute of Nephrology of Chinese PLA, Department of Nephrology, General Hospital of Chinese PLA, Medical School of Chinese PLA, Beijing 100853, China; pansai301@126.com (S.P.); chenpu301@126.com (P.C.); liujiaona301@126.com (J.L.); joker0104151687@126.com (W.L.); caiguangyan@sina.com (G.C.); jingtaopaian966@163.com (Z.Y.); wujie301@126.com (J.W.); tangli301@126.com (L.T.); wangyong301@263.net (Y.W.); shuweiduan@163.com (S.D.); 2 School of Electronic and Information Engineering, Beihang University, Beijing 100191, China; 17373540@buaa.edu.cn (Y.F.); xfwang@buaa.edu.cn (X.W.); daining@buaa.edu.cn (N.D.); jianglai.china@buaa.edu.cn (L.J.)

**Keywords:** multi-task learning, deep learning, immunofluorescence images, kidney

## Abstract

Chronic kidney disease is one of the most important causes of mortality worldwide, but a shortage of nephrology pathologists has led to delays or errors in its diagnosis and treatment. Immunofluorescence (IF) images of patients with IgA nephropathy (IgAN), membranous nephropathy (MN), diabetic nephropathy (DN), and lupus nephritis (LN) were obtained from the General Hospital of Chinese PLA. The data were divided into training and test data. To simulate the inaccurate focus of the fluorescence microscope, the Gaussian method was employed to blur the IF images. We proposed a novel multi-task learning (MTL) method for image quality assessment, de-blurring, and disease classification tasks. A total of 1608 patients’ IF images were included—1289 in the training set and 319 in the test set. For non-blurred IF images, the classification accuracy of the test set was 0.97, with an AUC of 1.000. For blurred IF images, the proposed MTL method had a higher accuracy (0.94 vs. 0.93, *p* < 0.01) and higher AUC (0.993 vs. 0.986) than the common MTL method. The novel MTL method not only diagnosed four types of kidney diseases through blurred IF images but also showed good performance in two auxiliary tasks: image quality assessment and de-blurring.

## 1. Introduction

Chronic kidney disease (CKD) is a non-communicable disease that contributes to high morbidity and mortality worldwide, including in China [1,2]. Recent studies have shown that air pollution significantly increases the risk and mortality of CKD [3,4]. Diabetes is the leading cause of CKD in developed countries, whereas glomerulonephritis predominantly causes CKD in Asia and developing countries [5,6,7]. Membranous nephropathy (MN) and IgA nephropathy (IgAN) are the most common forms of primary glomerulonephritis, whereas lupus nephropathy (LN) and diabetes nephropathy (DN) are the most common secondary types of glomerulonephritis in China [6,8]. These four pathological types contribute to more than 60% of all cases of CKD [9]. Therefore, it is imperative to identify and diagnose these four kidney diseases. Immunofluorescence (IF) is one of the most important methods used for the diagnosis of these four kidney diseases. By 2017, there were only 3.94 and 4.81 pathologists per 100,000 people in the United States and Canada, respectively [10]. This implies that many hospitals lack experienced pathologists to diagnose kidney diseases. Thus, it is necessary to develop an automatic classification system for kidney IF images that can identify the diagnosis of interest from a bank of IF images.

Digital pathology refers to the assemblage of digital workflow and imaging solutions that are geared towards creating a digital image-based practice environment [11]. Deep learning has been widely used in all aspects of social life, and the emergence of convolutional neural networks (CNN) has further expanded the application of artificial intelligence in digital pathology [11,12,13,14], which is expected to reduce delays or errors in diagnosis and treatment caused by insufficient pathologists [15]. Moreover, in some cases, the image diagnosis capabilities of CNN are far beyond that of humans [16,17]. A study showed that deep learning could diagnose DN based on only six types of IF staining [18]. There have also been studies that predicted systemic conditions and clinical metrics based on fundus photographs, suggesting that deep-learning algorithms can detect subtle associations that are undetectable to human observers [19,20]. 

In the image acquisition stage, the acquired images may be blurry due to human or instrumental factors, which makes image recognition and analysis difficult in most cases [21]. Most existing works were trained on non-blurred IF images [18,22]. Therefore, in this study, we proposed a novel multi-task learning (MTL) method to handle multiple tasks including the image quality assessment, de-blurring, and classification of blurred IF images. MTL is a learning paradigm in machine learning used to leverage the shared information and handle multiple correlated tasks. The effectiveness of MTL with CNN methods has recently been demonstrated in the medical field. The commonly used MTL method simply cascades tasks hierarchically. Notably, it only minimizes the mean square error (MSE) between the reconstructed and clear images in the de-blurring task, and it pays less attention to image semantic information that is beneficial for classification tasks [23,24]. To overcome this drawback, we proposed a novel MTL method named the MTL-IF method. We leveraged the well-trained classification subnet to guide the training stage of the de-blurring subnet. The results demonstrated that the MTL-IF method can improve the clinical diagnosis based on blurred IF images.

The main contributions of this study are as follows. (1) Novel image quality assessment and de-blurring methods are proposed to enhance the IF image quality, improve the accuracy, and improve the reproducibility of the classification model. (2) The proposed method has superior performance, as evidenced by its total accuracy of 0.94 and area under the receiver operating characteristic curve (AUC) score of 0.993 for blurred IF image classification.

## 2. Materials and Methods

### 2.1. Renal IF Image

Kidney specimens were obtained from patients undergoing renal biopsy treatment in the People’s Liberation Army General Hospital, Beijing, China. Tissue samples were quickly placed in liquid nitrogen, and the frozen tissue was cut into 3–4 μm thick sections in a cryostat. The corresponding mouse anti-human IgG, IgA, IgM, C3, C4, C1q, and Fibrinogen (Fib) fluorescein isothiocyanate antibody was used for labeling and staining. A fluorescence microscope (Olympus, Tokyo, Japan) was used to obtain IF images (200×). All images were taken following a standardized protocol and a 200 ms fixed exposure time.

### 2.2. Patients Data

We retrospectively collected IF images of patients with IgAN, MN, DN, and LN in our hospital from June 2003 to November 2013. Seven types of IF images—IgG, IgA, IgM, C3, C4, C1q, and Fib—were collected for each patient. The IF images of each patient were analyzed by two professional renal pathologists with over five years of work experience. When there were discrepancies in diagnosis, a third senior pathologist with over ten years of work experience intervened to make the correct diagnosis. The final diagnosis of kidney disease was made by nephrologists based on the patient’s medical history, examination results, and pathology including IF images, light microscopy images, and electron microscopy images. The inclusion criteria were: (1) primary IgAN, (2) primary MN, and (3) all DN and LN. The exclusion criteria were: (1) patients under 18 years old and (2) patients with two or more kidney diseases. We divided the data into training and test data in a ratio of 8:2. Meantime, in order to build a blurred IF image database to simulate the defocusing scene of microscope, all the IF images were blurred via the Gaussian method [25].

### 2.3. Image Quality Assessment and De-Blurring 

The image quality assessment (IQA) tasks take images as input and aims to regress the exacted feature into quality score. Specifically, our IQA subnet was composed of several convolutional layers for extracting features and linear layers for generating image quality scores. We set quality scores of 0 and 1 to represent blurred and non-blurred, respectively, a binary which is commonly adopted in the IQA tasks. Since the image quality assessment task was aimed to serve the de-blurring task, it did not need to assess the exact quality score of image; instead, it sonly roughly graded the IF image quality as blurred or non-blurred. Then, IF images needed to be de-blurred if the quality was judged as blurred. Otherwise, an image could be directly used for classification without de-blurring. The performance of image quality assessment was measured by the Pearson product-moment correlation coefficient (PLCC). The PLCC measures the correlation between predicted quality scores and ground truth scores, and it is commonly used in IQA tasks. Note that larger values indicate higher prediction correlations between IQA results and corresponding ground-truth. Specially, the PLCC metric can be formulated as follows:

$$PLCC=\frac{COV\left(X,Y\right)}{\delta_{X}\delta_{Y}}$$

In the above equation, X and Y denote the predicted quality results and the corresponding ground-truth quality scores vectors. $\delta_{X}$,$\delta_{Y}$ and COV(X,Y) denote the standard deviation and covariance of X and Y, respectively.

**Figure 1:**
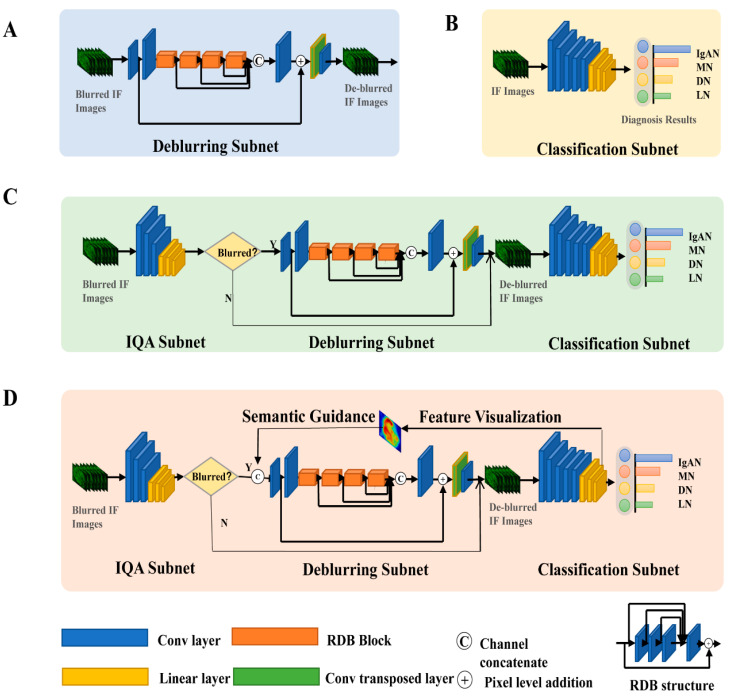
The framework of the network: (**A**) deblurring subnet, (**B**) classification subnet, (**C**) the common MTL method, and (**D**) the novel MTL-IF method. IQA: image quality assessment; IgAN: IgA nephropathy; MN: membranous nephropathy; DN: diabetic nephropathy; LN: lupus nephritis.

To de-blur blurred IF images judged by the image quality assessment subnet, we used a de-blurring subnet inspired by Zhao H et al. [26]. The main architecture of the de-blurring subnet contained five major components (shown in Figure 1A): shallow feature extraction, residual dense blocks (RDB), global feature fusion, global residual learning, and up-scaling. The forward process of the entire network can be briefly described as follows.

First, two convolutional layers are used to extract the shallow features, and then the extracted shallow features go through four RDB blocks. Each RDB block includes dense connection, local feature fusion, and local and residual connection. Dense connection refers to a direct connection of each convolutional layer to subsequent layers, which enhances local transmission and optimally uses features from all preceding layers. All local features are concatenated together and pass through a 1 × 1 convolutional layer to achieve local feature fusion. Global residual learning combines shallow features and global fusion features. Finally, the image is restored through up-scaling. The minimum mean square error mean square error (*MSE*) was used as the loss function, and Adam was used as the optimizer.

$$MSE=\frac{1}{mn}\overset{m-1}{\underset{i=0}{\sum }}\overset{n-1}{\underset{j=0}{\sum }}{\left|\left|I\left(i,j\right)-K\left(i,j\right)\right|\right|}^{2}$$

### 2.4. IF Image Classification Based on CNN

The fundamental AlexNet [27] was utilized as the main framework for classifying the IF images of four types of kidney diseases: IgAN, MN, DN, and LN. The framework of the AlexNet method consisted of convolutional layers for extracting features and linear layers for classifying (shown in Figure 1B). Due to the need for multiple stains when diagnosing per instance, we applied a multi-channel input mechanism. We leveraged the cross-entropy function as the loss function and Adam as the optimizer. The network was trained for 150 epochs with a batch size and learning rate of 64 and 0.0002, respectively.

### 2.5. Blurred IF Image Classification Using the Common MTL Method

The common MTL method simply cascaded the three aforementioned subnets by order. First, the IF images were input into the image quality assessment subnet to evaluate the image quality. If the images’ quality was judged as blurred, the images were input to De-blurrNet for de-blurring; otherwise, they were directly input to the classification subnet, and all IF images were input into the classification subnet in the end (shown in Figure 1C).

### 2.6. Classification of Blurred IF Image Using the Proposed MTL-IF Method

Since our ultimate goal was to improve the classification accuracy and clinical diagnosis of kidney diseases, we proposed the MTL method, which uses downstream high-level to guide low-level tasks. With the guidance of the classification task, the de-blurring subnet effectively focused on important regions in the classification task and paid less attention to unimportant regions or background. The main framework of the proposed multi-task learning model is shown in Figure 1D.

**Figure 2:**
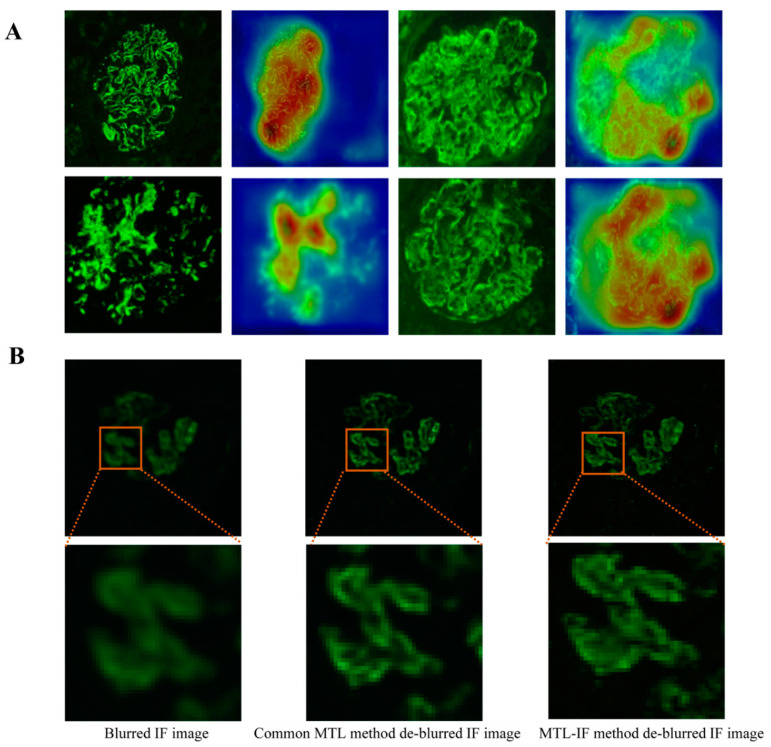
(**A**) The heat maps of IF images. (**B**) The de-blurred images generated by the common MTL method and the MTL-IF method, including the blurred IF image, common MTL method de-blurred IF image, and MTL-IF method de-blurred IF image.

To highlight the regions that were focused on by the classification task, we leveraged the Grad-CAM method proposed by Selvaraju et al. [28]. The Grad-CAM method uses class-specific gradient information flowing into the final convolutional layer of a CNN to produce a coarse localization map of the important regions in an IF image to guide de-blurring. Figure 2A shows the heat map of an IF image.

In the training phase, only the weights of the de-blurring subnet were updated by the error back-propagated, while the well-trained classification subnet based on clear IF images remained unchanged. First, the Grad-CAM method was used to visualize the feature map when blurred IF images were applied as input to the well-trained classification subnet. Second, the input blurred IF images combined with the corresponding feature maps in the channel-concatenate manner. Subsequently, the input images were deblurred, and MSE loss was used for updating de-blurring network. Finally, the deblurred images were re-entered into the AlexNet for classification. As a result, we updated the de-blurring network with an attention distribution that paid more attention to regions essential for clinical classification diagnosis.

### 2.7. Assessment of CNN Classification Performance

We tested some metrics, including recall, precision, and F1 score, to assess the classification performance of CNN. Finally, the receiver operating characteristic (ROC) curve was drawn, and the AUC was calculated. The chi-square test was used to compare the accuracy of different models. A *p*-value <0.05 was considered statistically significant. The aforementioned metrics are defined as follows.

Accuracy = (True Positives + True Negatives)/(True Positives + True Negatives + False Positives + False Negatives).

Accuracy is the ratio between the correct predictions and the total predictions.

Recall = (True Positives)/(True Positives + False Negatives).

Recall has the same statistical meaning as sensitivity.

Precision = (True Positives)/(True Positives + False Positives).

Precision has the same statistical meaning as the positive predictive value.

F1 score = 2 (Precision × Recall)/(Precision + Recall).

The F1 score is an indicator used in statistics to measure the accuracy of a classification model. The F1 score can be regarded as a weighted average of precision and recall. Its maximum value is 1, and its minimum value is 0.

## 3. Results

### 3.1. Image Data

We included 1608 patients’ IF images as the classifier dataset. Our IF diagnosis was jointly determined by three experienced doctors. If the IF images had no noticeable characteristics, our doctors combined light microscopy, electron microscopy, medical history, and our results to diagnose the diseases. There were 655, 348, 201, and 404 cases of IgAN, MN, DN, and LN, respectively. We divided the training and the test data into a ratio of 8:2. The training set had 1289 cases, with 523 cases of IgAN, 281 cases of MN, 162 cases of DN, and 323 cases of LN.

### 3.2. Image Quality Assessment and De-Blurring

The performance of the MTL-IF method was compared to that of the common method in two auxiliary tasks: image quality assessment and de-blurring. For the de-blurring task, the MSE metric was used to evaluate the performance. A lower MSE value indicated better de-blurring performance. The MSE of the proposed MTL method was decreased to 0.00115, whereas that of the common MTL method was decreased to 0.00147. Figure 2B shows the de-blurred images generated by the MTL-IF method.

### 3.3. Classification of Subnet Performance Based on Non-Blurred and Blurred IF Images

**Figure 3:**
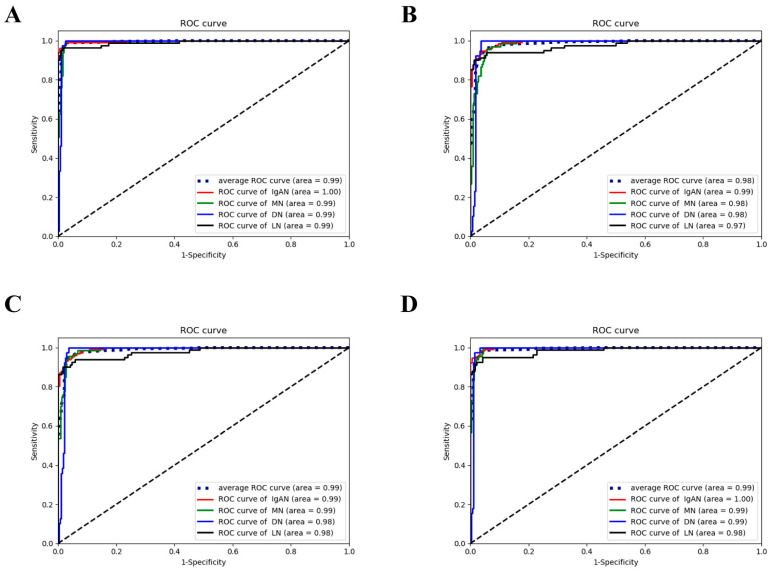
The ROC curve of each method. (**A**) ROC curve of non-blurred IF images test classification subnet; (**B**) ROC curve of blurred IF images test classification subnet; (**C**) ROC curve of blurred IF images test with the common MTL method; (**D**) ROC curve of blurred IF images test with the MTL-IF method. ROC: receiver operating characteristic; IgAN: IgA nephropathy; MN: membranous nephropathy; DN: diabetic nephropathy; LN: lupus nephritis.

The classification subnet was trained to identify IgAN, MN, DN, LN, and other kidney diseases. Table 1 shows the ability of the classification subnet to independently diagnose four diseases based on non-blurred IF images. The overall accuracy rate was 0.97, and the AUC was 0.995. For blurred IF images, the overall accuracy rate was 0.91 and the AUC was 0.982 (shown in Table 2). The ROC curve is shown in Figure 3. The accuracy of using blurred IF images to diagnose kidney disease significantly decreased.

### 3.4. Performance of the Two Methods in Disease Diagnosis Based on Blurred IF Images

The common MTL method improved the classification accuracy when applied to blurred IF images, with an overall accuracy rate of 0.93 and an AUC of 0.986 (shown in Table 3). The ROC curve is shown in Figure 3. The proposed MTL-IF method was more accurate than the common MTL method in diagnosing kidney disease when applied to blurred IF images. Its overall accuracy rate was 0.94 (*p <* 0.01), and the AUC was 0.993. The ROC curve is shown in Figure 3. Additional details regarding the performance of the two methods are shown in Table 3 and Table 4. Each disease was diagnosed at an average testing time of 0.57 s.

## 4. Discussion

AI has been widely used in the recognition of renal pathology [29], such as the recognition of cortical or medulla, glomeruli, renal tubules, and renal arteries [30,31], as well as the recognition of internal glomerular structures, such as the podocytes, mesangial cells, and mesangial area [32]. However, in the final diagnosis of kidney disease, the type, location, and shape of immune complex deposits are still important bases and have distinct characteristics, especially in IgAN and MN. The diagnosis of LN relies on medical history and autoantibody testing but cannot be directly diagnosed using light microscopy and IF unless the IF shows “full house”. The IF of DN has no noticeable characteristics, and its diagnosis depends on light microscopy, medical history, and peripheral small vessel disease, such as fundus vascular disease. However, deep learning can extract features that cannot be detected by human eyes. In our study, we used deep learning to mine the specific features of IF images to directly diagnose four types of kidney diseases. We utilized non-blurred IF images to train and test the classification subnet. The accuracy of diagnosing kidney disease using seven types of IF images was as high as 0.97, which suggested that CNN might identify not only distinctive IF images but also image features that the human eye misses. LN-V also manifests as the diffuse thickening of the glomerular basement membrane, similar to primary MN. Our method could distinguish these two diseases, indicating that the network captured features that the human eye did not pay attention to.

The non-standardization of image quality is major problem of AI classification and recognition, and it has led to the poor reproducibility of model. Any medical imaging technology involving human reports is subject to important explanatory subjectivity constraints. Poor reproducibility has been confirmed numerous times in previous renal histopathology studies [33], and the application of deep learning in renal pathology recently also confirmed it [22,31,34]. Several studies have incorporated multi-center pathological images to increase the range of model recognition images [35]. However, multi-center studies can only include as many types of pathological images as possible; they cannot include all types of pathological images. Additionally, multi-center research is often time-consuming and laborious. Therefore, it is particularly important to standardize the quality of images. In our study, to avoid the impact of different personnel and equipment on the image quality, we included an image quality assessment and de-blurring subnet. Our results showed that the de-blurring subnet significantly improved the clarity of blurred images. We believe that standardized pre-processing of IF images can be further utilized in the future, especially in retrospective designs.

The common MTL method, which simply cascades tasks by order, has been used to solve the problem of de-blurring and classification [36]. However, though this method minimizes the MSE between the reconstructed and non-blurred images, it does not address the semantic information that is beneficial to the classification tasks. It has been reported that using semantic information from high-level vision tasks to guide low-level vision tasks could achieve potential gains in high-level tasks [37]. Inspired by this strategy, we proposed the MTL-IF method. Compared with the common MTL method, the MTL-IF method significantly increased diagnosis accuracy when applied to blurred IF images and achieved comparable performance in two auxiliary tasks: image quality assessment and de-blurring. This demonstrated potential gains when applying the high-level task guidance. Moreover, the diagnosis speed of the proposed model was significantly faster than that of human eye recognition. According to reports, pathologist takes about 7.3 s to recognize and diagnose an IF image [22]. The time to recognize seven IF images is about 51.1 s. The MTL-IF model was found to be able to diagnose seven IF pictures in 0.57 s, so the MTL-IF model needs less time to recognize seven IF images than a pathologist.

## 5. Conclusions

This study shows that the MTL model can evaluate the quality of IF images of kidney disease and enhance the quality of low-quality images. It can also accurately classify and diagnose four types of kidney disease based on IF images. In the future, we hope to apply this model to identify other types of IF images of kidney diseases.

## Figures and Tables

**Table 1:** Classification subnet performance in disease diagnosis based on non-blurred IF images.

Diseases	Numbers	Accuracy	Recall	Precision	F1 Score	AUC
IgAN	132	0.98	0.98	0.98	0.98	0.997
MN	67	0.97	0.97	0.96	0.96	0.994
DN	39	0.95	0.95	0.93	0.94	0.992
LN	81	0.95	0.95	0.98	0.96	0.990
Total	319	0.97	0.96	0.96	0.96	0.995

IF: immunofluorescence; IgAN: IgA nephropathy; MN: membranous nephropathy; DN: diabetic nephropathy; LN: lupus nephritis; AUC: area under the curve.

**Table 2:** Classification subnet performance in disease diagnosis based on blurred IF images.

Diseases	Numbers	Accuracy	Recall	Precision	F1 Score	AUC
IgAN	132	0.87	0.87	0.98	0.92	0.992
MN	67	0.97	0.97	0.82	0.89	0.983
DN	39	0.92	0.92	0.86	0.89	0.983
LN	81	0.93	0.93	0.93	0.93	0.974
Total	319	0.91	0.92	0.90	0.91	0.982

IF: immunofluorescence; IgAN: IgA nephropathy; MN: membranous nephropathy; DN: diabetic nephropathy; LN: lupus nephritis; AUC: area under the curve.

**Table 3:** Common MTL method performance in disease diagnosis based on blurred IF images.

Diseases	Numbers	Accuracy	Recall	Precision	F1 Score	AUC
IgAN	132	0.90	0.90	0.98	0.94	0.993
MN	67	0.97	0.97	0.87	0.92	0.993
DN	39	0.92	0.92	0.90	0.91	0.983
LN	81	0.94	0.94	0.93	0.93	0.977
Total	319	0.93	0.93	0.92	0.92	0.986

IF: immunofluorescence; IgAN: IgA nephropathy; MN: membranous nephropathy; DN: diabetic nephropathy; LN: lupus nephritis; AUC: area under the curve.

**Table 4:** MTL-IF method performance in disease diagnosis based on blurred IF images.

Diseases	Numbers	Accuracy	Recall	Precision	F1 Score	AUC
IgAN	132	0.87	0.87	0.98	0.92	0.992
MN	67	0.97	0.97	0.82	0.89	0.983
DN	39	0.92	0.92	0.86	0.89	0.983
LN	81	0.93	0.93	0.93	0.93	0.974
Total	319	0.91	0.92	0.90	0.91	0.982

IF: immunofluorescence; IgAN: IgA nephropathy; MN: membranous nephropathy; DN: diabetic nephropathy; LN: lupus nephritis; AUC: area under the curve.

## Data Availability

All data are kept by the corresponding author.
